# Developmental Potency and Metabolic Traits of Extended Pluripotency Are Faithfully Transferred to Somatic Cells via Cell Fusion-Induced Reprogramming

**DOI:** 10.3390/cells11203266

**Published:** 2022-10-17

**Authors:** Jae-Hoon Song, Joonhyuk Choi, Yean-Ju Hong, Hyeonwoo La, Tae-Kyung Hong, Kwonho Hong, Jeong-Tae Do

**Affiliations:** 1 Department of Stem Cell and Regenerative Biotechnology, Konkuk Institute of Technology, Konkuk University, Seoul 05029, Korea; 2 3D Tissue Culture Research Center, Konkuk University, Seoul 05029, Korea

**Keywords:** cell fusion reprogramming, extended pluripotency, totipotency, metabolism, embryonic, extraembryonic

## Abstract

As a novel cell type from eight-cell-stage embryos, extended pluripotent stem cells (EPSCs) are known for diverse differentiation potency in both extraembryonic and embryonic lineages, suggesting new possibilities as a developmental research model. Although various features of EPSCs have been defined, their ability to directly transfer extended pluripotency to differentiated somatic cells by cell fusion remains to be elucidated. Here, we derived EPSCs from eight-cell mouse embryos and confirmed their extended pluripotency at the molecular level and extraembryonic differentiation ability. Then, they were fused with OG2^+/−^ ROSA^+/−^ neural stem cells (NSCs) by the polyethylene-glycol (PEG)-mediated method and further analyzed. The resulting fused hybrid cells exhibited pluripotential markers with upregulated EPSC-specific gene expression. Furthermore, the hybrid cells contributed to the extraembryonic and embryonic lineages in vivo and in vitro. RNA sequencing analysis confirmed that the hybrid cells showed distinct global expression patterns resembling EPSCs without parental expression of NSC markers, indicating the complete acquisition of extended pluripotency and the erasure of the somatic memory of NSCs. Furthermore, ultrastructural observation and metabolic analysis confirmed that the hybrid cells rearranged the mitochondrial morphology and bivalent metabolic profile to those of EPSCs. In conclusion, the extended pluripotency of EPSCs could be transferred to somatic cells through fusion-induced reprogramming.

## 1. Introduction

Cells are classified according to their potency, such as totipotency, pluripotency, multipotency, unipotency, and nullipotency. Totipotency, the ability to give rise to embryonic and extraembryonic lineages from a single cell, is possessed only by the fertilized one-cell embryo and 2- to 4-cell blastomere [1,2]. Therefore, totipotency exists transiently and cannot be maintained in stem cells in vitro. Pluripotent stem cells (PSCs) can be derived from the inner cell mass (ICM) of the blastocyst in vitro; these are called embryonic stem cells (ESCs) and retain the differentiation capacity shown in the epiblast of peri-implantation embryos [3,4]. PSCs can also be derived from reprogramming processes such as nuclear transfer, cell fusion, and transduction of reprogramming factors [5]. ESCs and other types of PSCs reprogrammed from somatic cells possess the developmental potency to give rise to all adult cell types, including germlines [6,7]. PSCs are further subdivided into naïve, formative, and primed PSCs according to their developmental/differentiation potentials and molecular properties, reflecting diverse aspects of early embryogenesis [4,8,9,10]. However, these PSCs still display a limited differentiation potential, and their potency is usually limited to embryonic lineages, somatic cells, and germ cells.

A new cell type, the extended pluripotent stem cell (EPSC), was recently generated from eight-cell-stage embryos using a chemically defined medium, the LCDM condition, supplemented with several chemicals that capture developmental potency by blocking cell fate decisions [11]. EPSCs display transcriptional and epigenetic profiles distinct from those of naïve or primed PSCs. Notably, EPSCs showed higher potential for differentiation into extraembryonic lineages in addition to embryonic lineages and a higher contribution in human–mouse interspecies chimeras than ESCs [11]. The expanded pluripotency of EPSCs allows the formation of blastoids (blastocyst-like structures composed of ICM and trophectoderm-like cells), providing a new possibility as an early embryonic developmental model and as a totipotent stem cell [12,13,14,15].

As mentioned previously, pluripotency can be obtained by reprogramming of differentiated somatic cells. Cell-fusion-mediated reprogramming is a widely known reprogramming method that rapidly converts differentiated somatic cells into pluripotent cells [5]. After cell fusion, the genome of differentiated cells, including somatic and extraembryonic cells, is reset by trans-acting reprogramming factors residing in PSC partners [16,17,18]. Various types of PSCs, such as ESCs, embryonic germ cells (EGCs), embryonic carcinoma cells (ECCs), and induced PSCs (iPSCs), have been demonstrated to induce successful reprogramming of somatic cells [19,20]. However, it remains unclear whether EPSCs can transfer extended pluripotency to somatic cells via cell fusion and whether the resulting hybrid cells can exhibit extended pluripotency.

In the present study, we report for the first time that EPSCs can transfer extended pluripotency to somatic cells via cell fusion. We established EPSC lines from eight-cell embryos and fused them with neural stem cells (NSCs), which are somatic stem cells. The resulting EPSC-NSC hybrid cells showed transcriptional characteristics similar to EPSCs and displayed dual potential for differentiation into both embryonic and extraembryonic lineages in both in vivo and in vitro differentiation systems. We further showed that cell-fusion-mediated reprogramming affects the metabolic phenotypes of somatic fusion partner cells, remodeling energy metabolism profiles to be similar to those of EPSCs. This study provides a new model for elucidating novel molecular reprogramming pathways to achieve extended pluripotency.

## 2. Materials and Methods

### 2.1. EPSC Cell Line Establishment and Culture

EPSCs were established from 8-cell embryos using previously described procedures [11]. Briefly, 2-cell embryos were recovered from 1.5 dpc pregnant BL6 female mice and developed to the 8-cell stage. Embryos were attached to mitotically inactivated C3H mouse embryonic fibroblasts (MEF) in LCDM/N2B27 medium. N2B27 medium consists of Dulbecco’s Modified Eagle Medium/Nutrient Mixture F-12 (DMEM/F12) (Gibco, Billings, MT, USA, 11320-033), Neurobasal (Gibco, 21103-049), 5% Knockout serum replacement (Gibco, 10828-028), N2 (Gibco, 17052-048), B27 (Gibco, 17504-044), 1X penicillin-streptomycin (P/S; Gibco, 15140-122), 1% non-essential amino acid (Gibco, 11040-050), and 0.1 mM β-mercaptoethanol (Gibco, 21985-023). An LCDM supplement consisting of 10^3^ U/mL human recombinant leukemia inhibitory factor (Millipore, Burlington, MA, USA, LIF1010), 3 mM CHIR99021(LC laboratories, Woburn, MA, USA, C-6556), 2 mM S-(+)-dimethindene maleate (Tocris, Bristol, Avon, UK, 1425), and 2 mM minocycline hydrochloride (Santa Cruz, Dallas, TX, USA, sc-203339) was added to the N2B27 medium before use. After 5–7 days, the outgrowths were dissociated into single cells using 0.25% trypsin-EDTA (Gibco, 25200-072) and passaged on new inactivated C3H MEF. The established EPSC cell lines were passaged on alternate days, and the culture medium was changed every day.

### 2.2. EGFP Transfection

The lentiviral EF1a-EGFP expression vector was used to insert ubiquitously expressed EGFP protein into EPSCs and ESCs. Lentivirus-containing EGFP vectors were generated by transfecting 293FT cells. Appropriate amounts of lentiviral EF1a-EGFP expression vectors, psPAX2 packaging vectors, and pMD2G envelope vectors were enclosed using Lipofectamine for 5 min. The enclosed vectors were then diluted in Opti-MEM for 20 min and transfected into 293FT cells for 6 h. Three days after transfection, the supernatants containing the virus were harvested.

One day before viral infection, 1 × 10^5^ ESCs were seeded on inactivated MEF. Then, 24 h after transduction, the infected EPSCs or ESCs expressing EGFP were passaged using 0.25% trypsin-EDTA, and EGFP+ single cells were manually picked to establish a homozygous cell line.

### 2.3. Cell Fusion and Hybrid Cell Culture

EPSCs and OG2^+/−^/ROSA26^+/−^ double-transgenic NSCs were washed with phosphate-buffered saline (PBS), dissociated into single cells, and then mixed at a 1:3 ratio (1 × 10^5^: 3 × 10^5^). The mixture was then centrifuged for 5 min at 400× *g* in a conical tube. The supernatant was removed thoroughly, and 1 mL of pre-warmed polyethylene glycol (PEG1500; Roche, Basel, Switzerland) was added to the cell pellet for 1 min. Then, 22 mL of Dulbecco’s modified Eagle’s medium (DMEM; Cytiva, Incheon, Korea, SH30021.01) was carefully added to the PEG–cell mixture with constant stirring. After centrifugation for 5 min at 400× *g*, the pellet was washed with PBS, gently resuspended in the LCDM condition, and incubated for 20 min at 37 °C in a 5% CO_2_ incubator. The mixture was then cultured in the LCDM condition. After 5–6 days, Oct4-GFP^+^ colonies were picked and attached to C3H MEF in LCDM. When the colonies expanded, they were dissociated into single cells using 0.25% trypsin-EDTA. To prevent contamination from non-fused cells, Oct4-GFP^+^ single cells were selected and homozygous cell lines were established. The established cell line was cultured in the same manner as the EPSC cell lines.

### 2.4. Cell Culture

ESCs were cultured on inactivated MEF, in a mouse ESC culture medium consisting of DMEM low glucose supplemented with 15% fetal bovine serum (FBS; Cytiva, Incheon, Korea), 0.1 mM non-essential amino acids, 1× penicillin/streptomycin/glutamine (P/S/G; Gibco, 10378-016), and 1 mM β-mercaptoethanol and 10^3^ U/mL leukemia inhibitory factor (Millipore, ESG1107).

NSCs were derived from heterozygous OG2^+/−^ ROSA^+/−^ strain, which was produced by crossing a homozygous ROSA26 X OG2 male and a wild-type female [21,22]. NSCs were cultured in NS medium in a gelatin-coated cell culture dish. The NS medium consisted of DMEM/F12 containing 7.5% bovine serum albumin (BSA; Gibco, 15260-037), 1X P/S/G, N2, epidermal growth factor (EGF; Gibco, PHG0311), and basic fibroblast growth factor (bFGF; R&D systems, Minneapolis, MN, USA, 233-FB-01M). Cells were passaged every 2–3 days, and the culture media were changed every 2 days.

### 2.5. Karyotype Analysis

The cells at 50% cell confluency were fixed in the metaphase for karyotyping by adding 3 µg/mL nocodazole to the culture medium for 16 h. The cells were then spun down at 200× *g* and treated with hypotonic (0.56%) KCl solution for 15 min. Next, the cells were fixed in a fixation buffer composed of 1:3 acetic acid and methanol and dropped onto a clean glass slide. The glass slides were air-dried and stained with 10% Giemsa solution (Sigma-Aldrich, St. Louis, MO, USA) for 15 min for microscopic observation.

### 2.6. In Vitro Random Differentiation

For random differentiation, cells were dissociated into single cells and suspended in a differentiation medium to form embryoid bodies (EBs) by the hanging drop method (800 cells per drop). The differentiation medium consisted of DMEM low glucose supplemented with 15% FBS, 1× P/S/G, 0.1 mM non-essential amino acids, and 1 mM β-mercaptoethanol. After 2 days, the formed EBs were harvested, washed, and attached to a 0.15% porcine-gelatin-coated culture dish in the medium. The medium was changed every two days, and differentiation proceeded for approximately 14–16 days.

### 2.7. In Vitro Extraembryonic Lineage Differentiation

EPSCs and hybrid cells were differentiated into extraembryonic endoderm stem (XEN) cells using a procedure modified from the differentiation protocol for ESCs [23]. First, cells were dissociated into single cells, and 1 × 10^5^ cells were seeded into inactivated MEF in converted XEN medium (standard XEN medium supplemented with all-trans retinoic acid (R&D systems, 0695) and activin A (Peprotech, Cranbury, NJ, USA, 120-14E)). After 3–4 days, the cells were dissociated into single cells using 0.25% trypsin-EDTA and passaged onto inactivated MEF-coated 4-well dishes in standard XEN medium. The standard XEN medium consisted of RPMI 1640 (Gibco, 11875-093), 15% FBS, 1X P/S, and 0.1 mM β-mercaptoethanol. Then, we picked morphologically similar colonies of XEN cells after 10–15 days, and the cells were passaged at 70–80% confluency. The culture medium was changed every alternate day.

The trophectoderm population was derived under trophoblast stem cell (TSC) medium and TSC-conditioned medium (TSC-CM). The TSC medium consisted of RPMI 1640 medium containing 20% FBS, 1X sodium pyruvate (Gibco, 11360-070), 1X P/S/G, and 0.1 mM β-mercaptoethanol and was supplemented with heparin (Sigma, St. Louis, MO, USA, H3393) and recombinant human FGF4 (Peprotech, 100-31). TSC-CM was prepared by incubating TSC medium without heparin and FGF4 on an inactivated MEF-coated dish for 3 days, followed by filtering. To differentiate EPSCs and hybrid cells, 1 × 10^5^ cells were attached to inactivated MEF in a mixture of TSC medium and TSC-CM (1:1 ratio). After two passages in the medium mixture, the cells were passaged in only TSC medium at a low density. The cells were further passaged at 5–60% confluence.

### 2.8. Chimeric Embryo Generation

The day before aggregation, 2-cell-stage mouse embryos were collected from 1.5 dpc B6C3F1 female mice and cultured in vitro in drops of G2 plus medium (Vitrolife, Gothenburg, Sweden, 10132) under OVOIL (Vitrolife, 10029) for 24 h. GFP-infected EPSCs or hybrid cells were passaged and seeded onto inactivated MEF. The following day, the cells were trypsinized, and clumps (4–10 cells per clump) were selected for aggregation with an 8-cell-stage mouse embryo with a denuded zona pellucida. Aggregated embryos were cultured overnight at 37 °C in a 5% CO_2_ incubator. After 24 h, the aggregated embryos were transferred into the left uterine horn of a 2.5 dpc pseudopregnant ICR recipient.

### 2.9. X-Gal Staining

The EPSC-NSC hybrid cells and chimeric embryos were stained with X-gal. For staining, cells were washed with PBS and fixed in 4% paraformaldehyde for 10 min at 4 °C. After washing with PBS three times, the cells were rinsed in rinsing solution: PBS containing 0.02% NP40 and MgCl_2_. The cells were stained with X-gal staining solution: rinsing solution supplemented with 25 µg/mL of 5-Bromo-chloro-3-indolyl-galactosidase (X-gal; Promega, Medison, WI, USA, V3941), 5 mM K3Fe(CN)_6_, and 5 mM K4Fe(CN)6. Cells were incubated at 37 °C and 5% CO2 for 24 h and visualized by light microscopy.

Chimeric embryos were fixed for 1 h and washed with PBS for 2–3 h at 4 °C. Embryos were then rinsed with rinsing solution for 1 h and stained with X-gal staining solution, followed by overnight incubation.

### 2.10. Electron Microscopy

For transmission electron microscopic (TEM) observations, the specimens were fixed in 4% paraformaldehyde and 2.5% glutaraldehyde in 0.1 M phosphate buffer (PB) for 3 h. After rinsing in 0.1 M PB, the samples were post-fixed in 1% osmium tetroxide for 30 min. The samples were then dehydrated in a graded ethanol series (50, 70, 80, 90, 95, and 100%). Polymerization of the infiltrated sample in Epon 812 was performed overnight at 60 °C.

Ultrathin sections were cut using an ultramicrotome (Leica Microsystems, Wetzlar, Germany) to a thickness of approximately 60–70 nm. The sectioned slices were collected on grids (200 mesh) and stained with 2% uranyl acetate and lead citrate. The prepared grids were examined using a transmission electron microscope (JEOL, Tokyo, Japan) operating at 60 kV.

### 2.11. Mitochondrial Length Measurement

Mitochondrial length and the maximum (Max)/minimum (Min) ratio of length were analyzed using electron microscopy images. Measurements were made using Image J 1.53 software (NIH), and over 29 mitochondria were measured per sample for data analysis.

### 2.12. Oxygen Consumption Rate Analysis

A Seahorse XFp analyzer was used to measure the oxygen consumption rate (OCR). Overall, 6 × 10^4^ cells of EPSCs, fusion hybrid cells, and 8 × 10^4^ cells of NSCs were cultured for 16 h after being seeded into a cell culture plate pre-coated with diluted Matrigel (Corning, NY, USA, 356234). For analysis, the medium was changed to XFp base media supplemented with D-glucose (Agilent, Santa Clara, CA, USA, 103577-100), sodium pyruvate (Agilent, 103578-100), and L-glutamine (Agilent, 103579-100). The OCR was measured using a Seahorse XFp analyzer (Seahorse Bioscience, Billerica, MA, USA). Measurement values were obtained after injection of oligomycin (1.5 µM), FCCP (0.1 µM), and rotenone/antimycin A (0.5 µM) (Agilent). The analysis was performed according to the manufacturer’s instructions.

### 2.13. Extracellular Acidification Rate Analysis

A Seahorse XFp analyzer was used to measure the extracellular acidification rate (ECAR). Overall, 6 × 10^4^ cells of EPSCs, fusion hybrid cells, and 8 × 10^4^ cells of NSCs were cultured for 16 h after being seeded into a cell culture plate pre-coated with diluted Matrigel. For analysis, the medium was replaced with XFp base medium supplemented with D-glucose, sodium pyruvate, and l-glutamine. The ECAR was measured using a Seahorse XFp analyzer (Seahorse Bioscience). Measurement values were obtained after injection of rotenone/antimycin A (0.5 µM) and 2-DG (80 mM) (Agilent Technologies, Clara, CA, USA). The analysis was performed according to the manufacturer’s instructions.

### 2.14. RNA Isolation and qRT-PCR

Total RNA was extracted from samples using TRIzol reagent according to the appropriate protocols, and the amount of RNA was measured using Nanodrop (Thermo Scientific, Waltham, MA, USA). Then, cDNA was synthesized from 1 μg extracted total RNA using SuperScriptTM III Reverse Transcriptase (Invitrogen, Waltham, MA, USA, 18080-044), Oligo(dT)12-18 Primer (Invitrogen, 18418-012), and 10 mM dNTP Mix (Invitrogen, 18427-013). Real-time polymerase chain reaction (real-time PCR) was carried out using TOPrealTM qPCR 2X PreMIX (Enzynomics, Daejeon, Korea, RT500M), and the results were analyzed using a Roche LightCycler 5480 (Roche). The primers used for real-time RT-PCR are listed in Table S1.

### 2.15. Immunocytochemistry

For immunocytochemistry, cells were fixed with 4% paraformaldehyde for 20 min at 4 °C. After washing with PBS, the cells were treated with 0.3% Triton X-100 in PBS for 10 min and blocked with PBS containing 3% bovine serum albumin (Bovogen, BSAS0.1) for 1 h at 25 °C. The cells were then treated with primary antibodies at the following concentrations: OCT4 (1:500, Santa Cruz Biotechnology, Dallas, TX, USA, SC-5279), NANOG (1:500, Abcam, Cambridge, UK, ab80892), EOMES (1:500, Abcam, ab23345), GATA4 (1:200, Abcam, ab84593), tubulin beta III isoform (TUJ1; 1:500, Millipore, Burlington, MA, USA, MAB1637), smooth muscle actin (SMA; 1:500, Abcam, ab7817), SOX17 (1:500, R&D Systems, AF1924), and CDX2 (1:1250, Abcam, ab76541). The following day, the primary antibodies were removed and the cells washed thrice with PBS for 10 min. Finally, the cells were labeled with fluorescence-conjugated secondary antibodies to detect the primary antibodies at the following concentrations: Alexa Fluor 488 (1:500) and Alexa Fluor 568 (1:500). Lastly, they were treated with DAPI or Hoechst in 0.3% Triton X-100 in PBS for 3 min at room temperature and washed.

### 2.16. Bulk RNA Sequencing

The total RNA concentration was calculated using Quant-IT RiboGreen (Invitrogen, #R11490). To assess the integrity of the total RNA, samples were run on a TapeStation RNA screentape (Agilent, #5067-5576). Only high-quality RNA preparations with RIN greater than 7.0 were used for RNA library construction.

A library was independently prepared with 1 μg of total RNA from each sample using the Illumina TruSeq Stranded mRNA Sample Prep Kit (Illumina, Inc., San Diego, CA, USA, #RS-122-2101). mRNA containing the poly A tail was purified using poly-T-attached beads. Following purification, the mRNA was fragmented into small pieces, and mRNA fragments were copied into first-strand cDNA using SuperScript II reverse transcriptase (Invitrogen, #18064014) and random primers. Second-strand cDNA was synthesized using DNA polymerase I, RNase H, and dUTP. Then, a single “A” base was added to cDNA fragments for adapter ligation. The products were purified and enriched by PCR to create the final cDNA library.

The libraries were quantified using KAPA Library Quantification kits for Illumina Sequencing platforms according to the qPCR Quantification Protocol Guide (KAPA BIOSYSTEMS, #KK4854) and qualified using TapeStation D1000 ScreenTape (Agilent Technologies, #5067-5582). Indexed libraries were then submitted to Illumina NovaSeq (Illumina, Inc., San Diego, CA, USA), and paired-end (2 × 100 bp) sequencing was performed by Macrogen Inc. (Seoul, Korea).

### 2.17. Post Sequencing Data Analysis

For the raw sequencing reads, adapter trimming was performed with a skewer (v0.2.2) [24] with the -L 100 and -e options. The output file was mapped to the mm10 UCSC Mus musculus genome using the STAR (v2.7.9a) [25] read aligner. The results from STAR were quantified and normalized to FPKM values using the Cuffnorm of the Cufflinks (v2.2.1) [26] toolset.

The quantified data were further analyzed and visualized using R (v4.2.0) and its affiliated packages. A two-dimensional principal component analysis (PCA) plot was produced using ggplot2 (v3.3.6) [27]. The corrplot (v0.92) [28] package was used for the correlation plot. In the comparison using the scatter plot, genes having an average expression level greater than FPKM3 and a fold change greater than 3 were defined as differentially expressed genes (DEGs). A scatter plot was produced using the plot function in R. In the expression analysis by the heatmap, DEGs were defined as genes with expression levels greater than 3 in all samples and a fold change greater than 3 in comparison between any two samples. Heatmaps and the hierarchical clustering of genes were produced using the heatmap2 function of the gplot (v3.1.3) [29] package. Gene ontology analysis was performed using DAVID (v2021update) [30,31], and the associated gene lists for specific gene ontology terms were acquired from DAVID’s Knowledgebase. Data for each sample are available at the Gene Expression Online under Accession Number GSE210520.

### 2.18. Statistical Analysis

All experiments were performed in triplicate, except for bulk RNA sequencing, which was performed in duplicate. Experimental data are presented as the mean ± standard deviation (SD). The significance of differences between sample data was evaluated using Student’s t-test or one-way analysis of variance (ANOVA) in SAS software version 9.4 (SAS Institute Inc., Cary, NC, USA). Tukey’s post hoc test was used for multiple comparisons. For mitochondrial morphology analysis, Dunn’s post hoc test was used. Statistical significance was set at *p* < 0.05.

### 2.19. Animal Use Ethical Statement

All methods used in this study were carried out in accordance with national animal care and use guidelines laws, and all experimental protocols were approved by the Institutional Animal Care and Use Committee of Konkuk University.

## 3. Results

### 3.1. Derivation and Characterization of Extended Pluripotent Stem Cells

**Figure 1:**
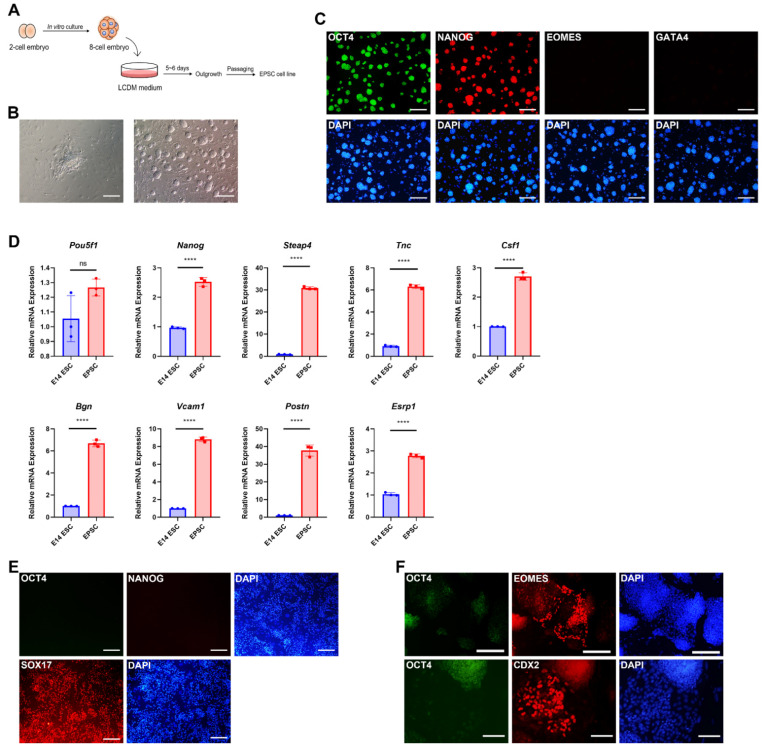
Establishment and characterization of extended pluripotent stem cells (EPSCs). (**A**) Schematic illustration for the establishment of EPSC lines from 8-cell embryos. (**B**) Representative images of 8-cell-stage embryo 5 days after seeding (left) and established EPSCs at passage 3 (right). Scale bars: 200 μm. (**C**) Immunocytochemistry images of EPSCs. EPSCs are shown to express pluripotency markers OCT4 and NANOG, whereas extraembryonic markers EOMES and GATA4 are not expressed. Scale bars: 200 μm. (**D**) quantitative RT-PCR analysis of EPSCs and ESCs about EPSC-specific upregulated genes. Data are presented as the mean ± SD for *n* = 3 independent experiments and every dot indicates the expression value of each sample. Gapdh was used as a housekeeping gene. EPSCs displayed a strong expression of genes that are overexpressed in EPSCs compared to ESC, as previously published. Student’s *t*-test: ns, non-significant; **** *p* < 0.0001. (**E**,**F**) Immunochemistry images of XEN (**E**) and TSC populations (**F**) directly converted from EPSCs. EPSC-derived XEN cells express primitive endoderm marker SOX17 (red) without expression of pluripotency marker OCT4 (green). Scale bars: 200 μm. TSCs converted from EPSCs were shown to express EOMES and CDX2 (red), but not OCT4 (green). Nuclei were stained by DAPI (blue). Scale bars: 200 μm (EOMES), 100 μm (CDX2).

EPSCs were established from C57BL/6 mouse eight-cell embryos using LCDM as previously described by Yang et al. [11] (Figure 1A). Five to six days after seeding, outgrowths were observed around the seeded embryos. Round and dome-like colonies were formed after passaging the outgrowths two–three times. These EPSCs could be stably cultured for over 40 passages without morphological changes or differentiation (Figure 1B). Immunocytochemistry analysis showed that the established EPSCs expressed pluripotency markers such as OCT4 and NANOG, whereas extraembryonic lineage markers such as EOMES and GATA4 were not expressed (Figure 1C).

Next, we determined several molecular characteristics of EPSCs that were distinguishable from those of ESCs. Typically, EPSCs show differences in the transcriptional profile and developmental potential both in vitro and in vivo [11,32]. First, we analyzed the transcriptomic characteristics of EPSCs using real-time qPCR. We checked Steap4, Tnc, Csf1, Bgn, Vcam1, Postn, and Esrp1, which were suggested to be differentially expressed between EPSCs and ESCs in Yang et al.’s previous report [11]. Real-time qPCR analysis indicated that EPSCs have different transcriptional expression patterns from ESCs, which is consistent with previously published EPSC transcriptome data (Figure 1D). Subsequently, we sought to identify the dual potential of EPSCs toward embryonic and extraembryonic lineages in vivo and in vitro. In vitro differentiation through embryoid body (EB) formation indicated lineage commitment to all three germ layers (Figure S1). Next, we examined the lineage conversion ability of EPSCs. EPSCs can be directly converted into extraembryonic stem cell lineages such as extraembryonic endoderm (XEN) cells and trophoblast stem cells (TSCs) [1,11]. As expected, EPSCs were efficiently converted into XEN cells (SOX17^+^) and TSCs (EOMES^+^ and CDX2^+^), which did not express pluripotency markers (Figure 1E,F). These data demonstrated that newly derived EPSCs have distinct molecular and developmental features compared to ESCs, which is consistent with several previous studies.

### 3.2. Extended Potential Reprogramming of Somatic Cells by Cell Fusion with EPSCs

**Figure 2:**
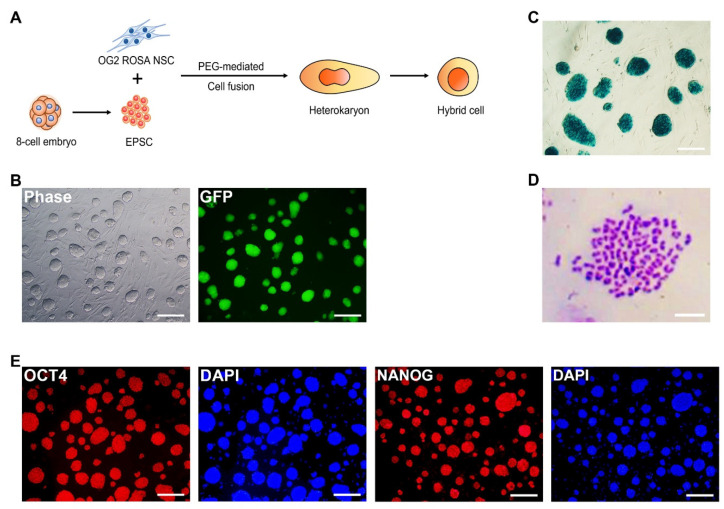

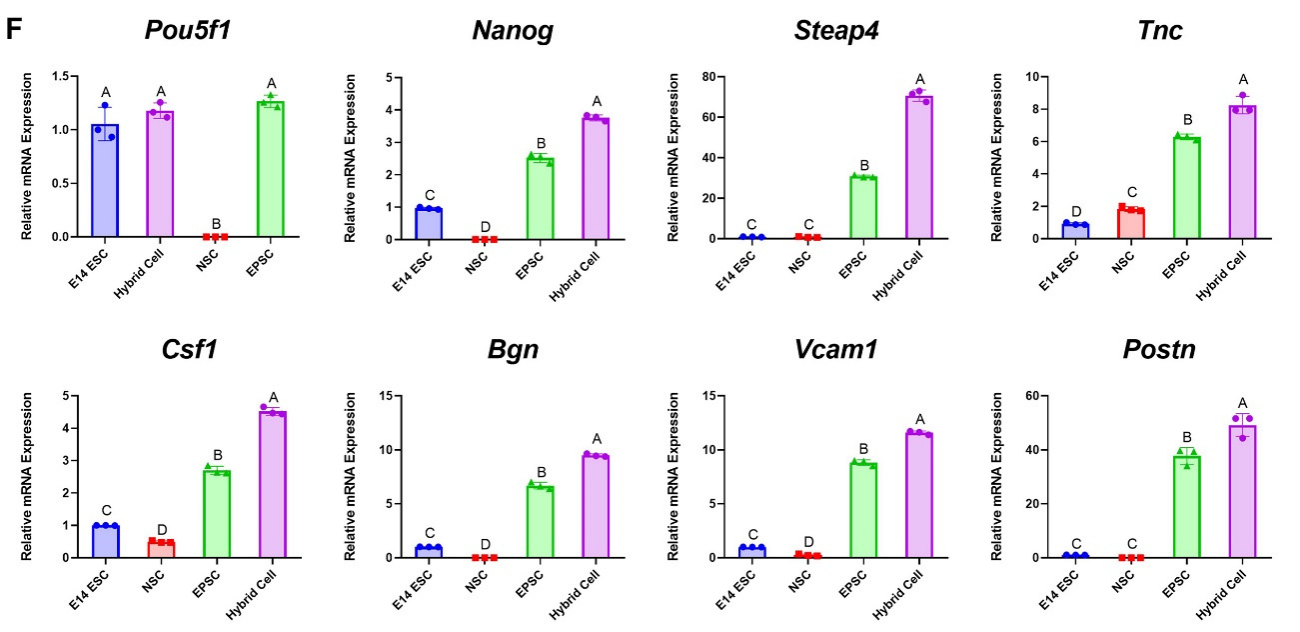
Reprogramming of NSCs by cell fusion with EPSCs and characteristics of the fusion hybrid cells. (**A**) Schematic illustration of cell-fusion-mediated reprogramming of NSCs with EPSCs. (**B**) Bright-field image (left) and fluorescence image (right) of Oc4-GFP^+^ established EPSC-NSC hybrid cells. Scale bars: 200 μm. (**C**) X-gal staining assay of EPSC-NSC hybrid cells. Scale bars: 200 μm. (**D**) Karyotype analysis showed the near-tetraploidy of EPSC-NSC hybrid cells. Scale bars: 50 μm. (**E**) Immunocytochemistry images of EPSC-NSC hybrid cells for pluripotency markers, OCT4 and NANOG (red). Nuclei were counterstained by DAPI (blue). Scale bars: 200 μm. (**F**) quantitative RT-PCR analysis of EPSC-NSC hybrid cells about EPSC-specific upregulated genes. Data are presented as the mean ± SD for *n* = 3 independent experiments and every dot indicates the expression value of each sample. Gapdh was used as a housekeeping gene. EPSC-NSC hybrid cells expressed similar level of Oct4, Nanog, Tnc, Bgn, Vcam1, and Postn to EPSCs. Hybrid cells expressed higher levels of Steap4 and Csf1 than EPSCs. EPSCs and EPSC-NSC hybrid cells showed a higher level of EPSC markers, Steap4, Tnc, Csf1, Bgn, Vcam1, and Postn. ^A–D^ Uppercase indicates significant differences among different groups at *p* < 0.0001. Data were analyzed using one-way ANOVA and Tukey’s post hoc with SAS^®^ software, version 9.4 (Institute of INC, Cary, NC, USA).

To test whether EPSCs can transfer extended pluripotency to somatic cells, we fused EPSCs with NSCs to induce fusion-induced reprogramming. To monitor the reprogramming of NSCs, we used OG2^+/−^/ROSA26^+/−^ double-transgenic NSCs, which express GFP under the control of the distal enhancer of OCT4 expression and ubiquitously express lacZ genes [33]. NSCs and EPSCs were fused using the PEG-mediated method [22] (Figure 2A). Oct4-GFP^+^ colonies were observed after 2 days of fusion, and Oct4-GFP^+^ cells, which were expected to be EPSC-NSC hybrids, were stably maintained over 40 passages (Figure 2B). As Oct4 expression is restricted to PSCs and germ cells [34,35], Oct4-GFP^+^ cells should be reprogrammed cells by fusion with EPSCs. Oct4-GFP^+^ cells were also positive for the X-gal staining (Figure 2C). Karyotype analysis demonstrated that the cells exhibited near-tetraploidy (4N) (Figure 2D). Meanwhile, OG2^+/−^/ROSA26^+/−^ NSCs cultured in the LCDM condition ceased to proliferate, confirming that the reprogramming of NSCs was not driven by the culture conditions (Figure S2). Taken together, these results indicate that Oct4-GFP^+^ cells are tetraploid hybrid cells that are successfully reprogrammed by cell fusion between NSCs and EPSCs. 

Subsequently, we investigated whether the hybrid cells obtained the characteristics of extended pluripotency. Immunocytochemistry analysis confirmed that Oct4-GFP^+^ hybrid cells expressed pluripotency markers OCT4 and NANOG (Figure 2E). We then compared the expression patterns of EPSC-specific genes such as Steap4, Tnc, Csf1, Bgn, Vcam1, and Postn. The expression levels of these EPSC-specific genes were similar between EPSCs and EPSC-NSC hybrid cells, but the expression levels of ESCs and NSCs were significantly lower than those of EPSCs (Figure 2F). Interestingly, the expression levels of EPSC-specific genes in the hybrid cells were higher than those in EPSCs (Figure 2F). These results demonstrate that somatic cells can be reprogrammed into an extended pluripotent state by fusion with EPSCs at the transcriptional level.

### 3.3. Developmental Potency of Hybrid Cells into Both Embryonic and Extraembryonic Lineages

**Figure 3:**
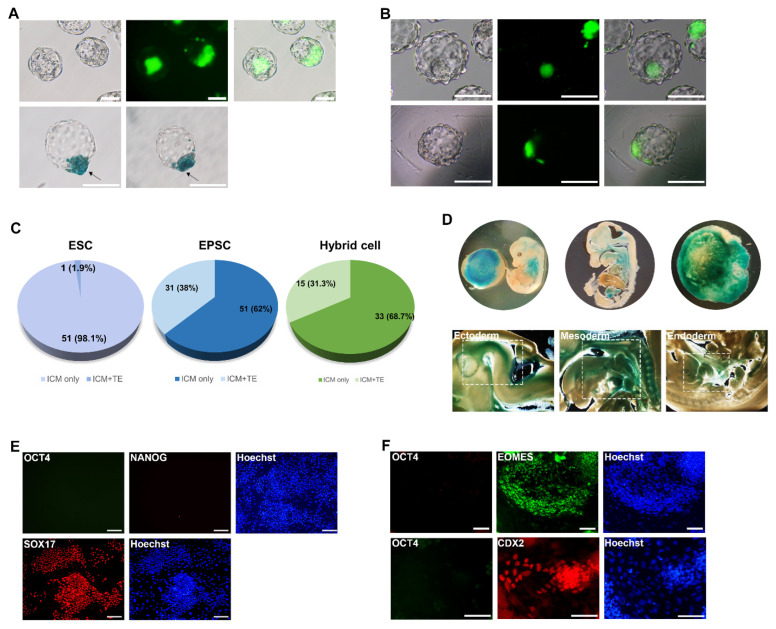
In vivo and in vitro differentiation potential of EPSC-NSC hybrid cells. (**A**) Images of chimeric blastocysts formed by aggregation of morular and EPSC-NSC hybrid cells. Bright-field and green fluorescence images (upper panel) and X-gal staining images (lower panel) of E4.5 chimeric blastocysts. Arrows indicate the X-gal-positive trophectoderm area of chimeric blastocysts. Scale bars: 100 μm. (**B**) Images of chimeric blastocysts formed by the aggregation of morular and ESCs (upper panel) or EPSCs (lower panel). ESCs and EPSCs expressing GFP ubiquitously were used for chimeric blastocyst formation to trace the cell fate. The contribution to trophectoderm was observed in chimeric blastocysts formed by EPSCs, but rarely formed by ESCs. Scale bars: 100 μm. (**C**) Contribution rate to ICM only or both ICM and trophectoderm (TE) in chimeric blastocysts formed by aggregation of ESCs, EPSCs, and EPSC-NSC hybrid cells with morula-stage embryos. (**D**) X-gal staining images of E13.5 chimeric embryos formed from chimeric blastocysts (using EPSC-NSC hybrids); entire embryo (upper left), a sectioned embryo (upper middle), and placenta (upper right). X-gal-positive cells (hybrid contribution) are shown in ectodermal, mesodermal, and endodermal tissues of the sectioned E13.5 chimeric embryos formed from chimeric blastocysts (using EPSC-NSC hybrids). Scale bars: 500 μm. (**E**,**F**) Immunocytochemistry images of XEN cells and TSC populations directly converted from EPSC-NSC hybrid cells. (**E**) XEN cells from hybrid cells express representative primitive endoderm marker SOX17 (red) without pluripotency marker OCT4 (green). Scale bars: 200 μm. (**F**) TSCs derived from hybrid cells were shown to express EOMES and CDX2 (red), but not OCT4 (green). Scale bars: 100 μm.

One of the distinct features of EPSCs is their expanded differentiation potency toward not only embryonic, but also extraembryonic lineages [11]. If NSCs obtained extended pluripotency through fusion with EPSCs, EPSC-NSC hybrid cells could functionally recapitulate the differentiation potency of EPSCs similar to obtaining pluripotency after fusion with pluripotent stem cells [5]. Thus, we attempted to verify whether hybrid cells could differentiate into both embryonic and extraembryonic lineages under in vivo and in vitro conditions [5]. First, we verified the in vivo developmental potential of reprogrammed EPSC-NSC hybrid cells using chimera formation analysis. Chimeric blastocysts were generated by the aggregation of morula and EPSC-NSC hybrid cells and further cultured to the blastocyst stage (Figure 3A). As a control experiment, chimeric blastocysts were generated using ESCs and EPSCs, which ubiquitously express the GFP transgene (Figure 3B). EPSC-NSC hybrid cells, ESCs, and EPSCs successfully formed chimeric blastocysts, in which aggregated cells were integrated into the ICM (Figure 3A,B). Notably, a contribution to the trophectoderm was observed in chimeric blastocysts formed by EPSCs and hybrid cells, but rarely in those formed by ESCs. To check the extraembryonic contribution, we counted the blastocysts showing the extraembryonic contribution (trophectoderm) by fluorescence expression (for EPSCs and ESCs) or X-gal staining (for hybrid cells). Chimeric blastocysts formed using EPSCs and reprogrammed hybrid cells showed a high ratio of extraembryonic contribution (38% and 31.3%, respectively; Figure 3C), whereas those formed using ESCs rarely contributed to trophectoderm formation (1.9%) (Figure 3C). Next, we investigated the developmental potential of reprogrammed hybrid cells in 13.5 dpc chimeric embryos (Figure 3D). Notably, EPSC-NSC hybrid cells contributed to the placenta and various body tissues in 13.5 dpc chimeric embryos (Figure 3D). In the X-gal-stained 13.5 dpc embryos, X-gal-positive cells, which originated from hybrid cells, were detected in the placenta and all three germ layer tissues: ectoderm (brain), mesoderm (heart), and endoderm (intestine) (Figure 3D). These results suggest that reprogrammed hybrid cells display extraembryonic developmental potential, as observed in EPSCs.

Next, we investigated whether EPSC-NSC hybrid cells could be converted into extraembryonic stem cell lineages, TSCs, and XEN cells, in vitro, which is the distinct feature of EPSCs that distinguishes them from conventional pluripotent stem cells [11,36]. During in vitro differentiation through EB formation, we examined CDX2-positive cells in differentiating EPSCs and hybrid cells, but these were rarely examined in differentiating ESCs (Figure S3B). After differentiation in XEN cell-derivation medium, hybrid cells were converted into XEN cells, which were positive for SOX17 without the expression of pluripotency markers such as OCT4 and NANOG (Figure 3E). On the other hand, under TSC-derivation conditions, hybrid cells could also be converted into TSCs expressing EOMES and CDX2 without pluripotency marker expression (Figure 3F). Taken together, these results suggest that EPSC-NSC hybrid cells can recapitulate the in vivo and in vitro developmental potency of EPSCs.

### 3.4. Global Gene Expression Patterns of EPSCs, ESCs, NSCs, and EPSC-NSC Hybrid Cells

**Figure 4:**
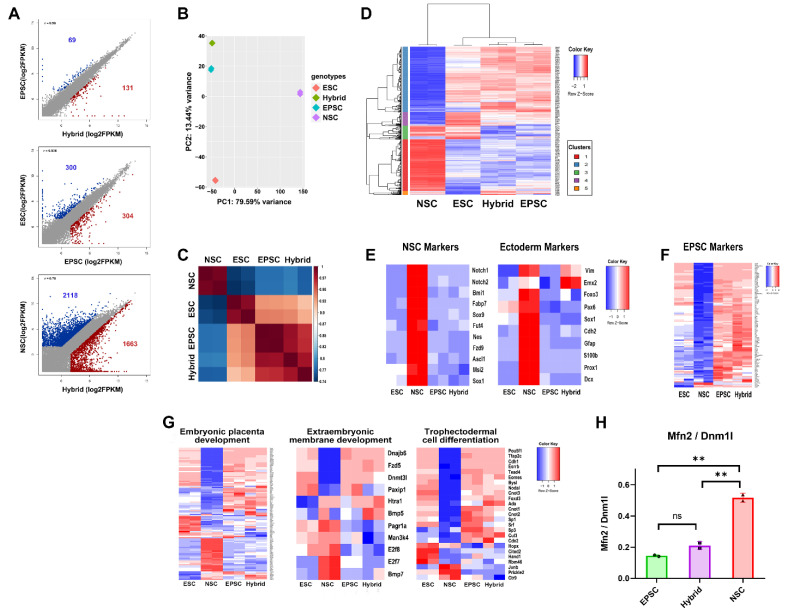
RNA sequencing analysis of ESCs, EPSCs, NSCs, and EPSC-NSC hybrid cells. (**A**) Scatter plot analysis showing the pairwise comparisons between EPSCs and hybrid cells, ESCs and EPSCs, and NSCs and hybrid cells. (**B**) Two-dimensional PCA analysis of ESCs, EPSCs, NSCs, and EPSC-NSC hybrid cells. (**C**) Correlation matrix analysis of ESCs, EPSCs, NSCs, and EPSC-NSC hybrid cells. (**D**) Hierarchical heatmap cluster of DEGs among ESCs, EPSCs, NSCs, and EPSC-NSC hybrid cells. (**E**) Heatmap of expression of ectoderm (left) and NSC marker genes (right) in ESCs, EPSCs, NSCs, and EPSC-NSC hybrid cells. (**F**) Heatmap of expression of EPSC marker genes in ESCs, EPSCs, NSCs, and EPSC-NSC hybrid cells. (**G**) Heatmap of expression of genes related to placenta development in ESCs, EPSCs, NSCs, and EPSC-NSC hybrid cells. (**H**) Ratio of *Mfn2/Dnm1l* in EPSCs, NSCs, and EPSC-NSC hybrid cells. Data are presented as the mean ± SD for *n* = 2 independent experiments and every dot indicates the expression value of each sample. Student’s *t*-test: ns, non-significant, ** *p* < 0.01.

We performed RNA sequencing analysis and compared the global gene expression patterns of the EPSCs, NSCs, and EPSC-NSC hybrid cells. The global gene expression patterns of EPSC-NSC hybrid cells were very similar to those of EPSCs; only 200 genes were differentially expressed, in comparison with the 3781 genes for EPSC-NSC hybrid cells vs. NSCs and 604 genes for EPSCs vs. ESCs (FPKM > 2, fold change > 2). Among the 200 genes differentially expressed between hybrid cells and EPSCs, 69 genes were overexpressed in EPSC, whereas 131 genes were abundantly expressed in EPSC-NSC hybrid cells (Figure 4A). The PCA plot and correlation matrix analysis revealed that the overall gene expression profile of hybrid cells was distinct from that of ESCs and NSCs, but similar to that of EPSCs (Figure 4B,C). Furthermore, the Gene Ontology-biological process (GO: BP) analysis revealed that both EPSCs and EPSC-NSC hybrid cells, compared to ESCs, overexpressed in terms of “response to mechanical stimulus”, “collagen fibril organization”, and “cell differentiation”. whereas they were downregulated in “multicellular organism development”, “defense response to virus”, and “negative regulation of viral genome replication” (Figure S4A). There were 2070 of the DEGs that were clustered into five hierarchical clusters by GO:BP analysis (Figure 4D and Figure S4B). Notably, clusters 2 and 3 showed the upregulated and downregulated gene sets, respectively, in both EPSCs and hybrid cells. EPSCs and hybrid cells clustered together and were highly enriched in terms associated with “translation” and “RNA processing”. However, genes enriched in NSCs, such as “biometabolic and biosynthetic process of lipid, steroid, fatty acid, and cholesterol” and “axonogenesis”, were not observed in reprogrammed fusion hybrid cells (Figure 4D and Figure S4B). These results indicate that EPSC-NSC hybrid cells successfully acquired the transcriptional characteristics of EPSCs after cell-fusion-induced reprogramming.

Next, we verified whether the transcriptional memory of NSCs was erased after reprogramming by cell fusion. Reprogrammed hybrid cells no longer expressed early ectodermal and NSC markers (Figure 4E and Figure S5), whereas they expressed EPSC-specific markers (Figure 4F), suggesting that the NSC genome was successfully reprogrammed and the somatic transcriptional memory completely erased after fusion with EPSCs.

Furthermore, we compared the differences in expression patterns for developmental potential toward extraembryonic trophoblast lineages among EPSCs, NSCs, ESCs, and EPSC-NSC hybrid cells (Figure 4G and Figure S6). EPSCs and EPSC-NSC hybrid cells showed expression patterns similar to those of genes associated with embryonic placental development, extraembryonic membrane development, and trophectodermal differentiation. These results may explain the favorable differentiational/developmental potency of EPSCs and EPSC-NSC hybrid cells to the extraembryonic lineage (placental contribution in the chimera assay and conversion ability to TSCs). Consequently, we demonstrated that the somatic memory of NSCs was erased and reprogrammed to the state of an extended pluripotent fusion partner through cell–cell fusion.

Lastly, we assessed the transcriptional similarity between EPSCs and hybrid cells by analyzing the genes associated with differentiation and mitochondrial dynamics. We previously devised an index to measure the extent of differentiation using the expression levels of the mitochondrial-dynamics-related genes *Mfn2* and *Dnml1* [37]. The *Mfn2*/*Dnm1l* ratio was relatively higher in more differentiated cells. For example, the *Mfn2*/*Dnm1l* ratio in differentiated somatic cells is higher than that in pluripotent stem cells. Consistently, the *Mfn2*/*Dnm1l* ratio in NSCs was much higher than that in EPSCs (Figure 4H). Notably, EPSC-NSC hybrid cells showed a similar *Mfn2*/*Dnm1l* ratio to EPSCs (Figure 4H), indicating that EPSCs and hybrid cells have the same extent of differentiation.

### 3.5. Mitochondrial Dynamics of EPSC-NSC Hybrid Cells Recapitulate That of EPSCs

**Figure 5:**
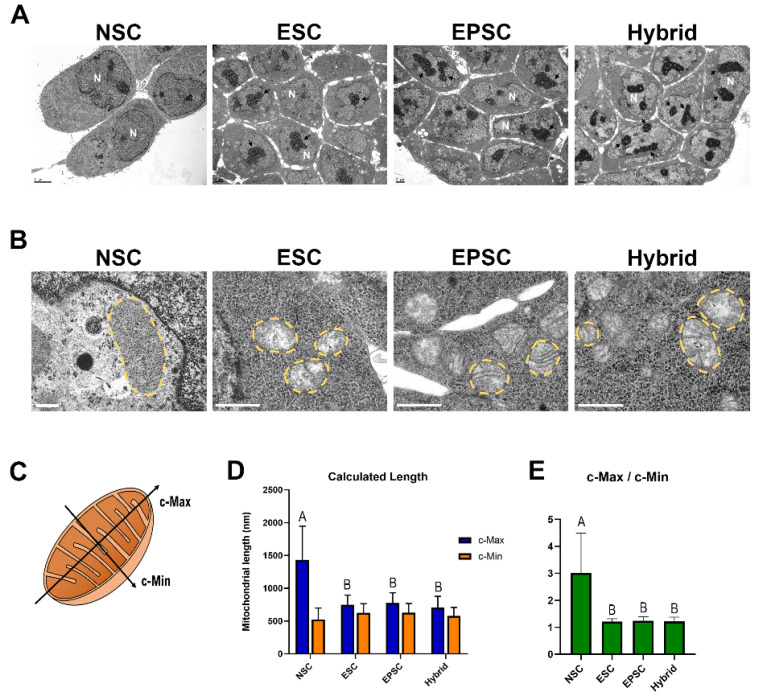
Analysis of mitochondrial morphology among ESCs, NSCs, EPSCs, and EPSC-NSC hybrid cells. (**A**) Representative images of the intracellular ultrastructure of each cell type were observed using transmission electron microscopy (TEM). Black arrows indicate the nucleoli in the nuclei of each cell type. Nuclei (N). Scale bars: 2 μm. (**B**) Representative images of mitochondria of each cell type observed through transmission electron microscopy (TEM). Yellow dotted lines indicate the morphology of mitochondria. Scale bars: 0.4 μm (NSC), 1 μm (ESCs, EPSCs, hybrid cells). (**C**) Illustration of the criteria for the measurement of mitochondrial axes’ length. (**D**) Analyzed mitochondrial length of each axis using the criteria. Data are presented as the mean ± SD for 29 independent mitochondrial samples. ^A,B^ Uppercase indicates significant differences among different groups at *p* < 0.0001. Data were analyzed using one-way ANOVA and Dunn’s post hoc with SAS^®^ software, version 9.4, (Institute of INC, Cary, NC, USA). (**E**) The ratio of the c-Max and c-Min axes of mitochondria. Data are presented as the mean ± SD for 29 independent mitochondrial samples. ^A,B^ Uppercase indicates significant differences among different groups at *p* < 0.0001. Data were analyzed using one-way ANOVA and Dunn’s post hoc with SAS^®^ software, version 9.4, (Institute of INC, Cary, NC, USA).

In addition to transcriptional dynamics, intracellular organelles are also remodeled during the reprogramming process [38]. Therefore, we compared the morphology of the nucleus and mitochondria of ESCs, NSCs, EPSCs, and EPSC-NSC hybrid cells using transmission electron microscopy (TEM). ESCs and EPSCs have a relatively higher nucleus-to-cytoplasm ratio than NSCs (Figure 5A), which is consistent with previous reports that pluripotent stem cells have a higher nucleus-to-cytoplasm ratio than differentiated cells [39]. There were no significant differences between EPSCs and ESCs. Notably, EPSC-NSC hybrid cells also showed a nucleus-to-cytoplasm ratio similar to that of EPSCs and ESCs (Figure 5A), indicating the recapitulation of mitochondrial dynamics during fusion-induced reprogramming. Moreover, EPSC-NSC hybrid cells showed multiple nucleoli with denser nucleolar structures than diploid cells, which are typical characteristics of tetraploid cells (Figure 5A).

Next, we compared the mitochondrial morphology in each cell type. ESCs showed globular mitochondria with poorly developed cristae, as expected. Meanwhile, NSCs had relatively elongated, rod-like mitochondria with dense, well-developed cristae structures (Figure 5B). The mitochondrial morphology of EPSCs was globular, with immature cristae, similar to that of ESCs (Figure 5B). Given that mitochondrial morphology is maintained as globular during embryogenesis until the preimplantation stage [40], it is reasonable to say that EPSCs derived from the eight-cell stage also have the same mitochondrial morphology. The mitochondrial morphology of NSCs was remodeled into immature and round pluripotency-like states during reprogramming by fusion with EPSCs (Figure 5B). Next, we measured the “calculated-maximum (c-Max)” and “calculated minimum (c-Min)” to more accurately analyze the mitochondrial morphology (Figure 5C–E). The mean values of “c-Max/c-Min” in ESCs (1.21) and EPSCs (1.24) were almost the same, while those of NSCs (3.02) were much higher (Figure 5C–E). As expected, the mean value of “c-Max/c-Min” in the EPSC-NSC hybrid cells (1.22) was almost the same as that in ESCs and EPSCs (Figure 5C–E). Taken together, these results indicate that reprogramming to extended pluripotency by cell fusion entails the complete remodeling of intracellular organelles.

### 3.6. Bioenergetic Metabolism Profiles of NSCs Were Remodeled to the State of EPSCs after Reprogramming by Cell Fusion with EPSCs

**Figure 6:**
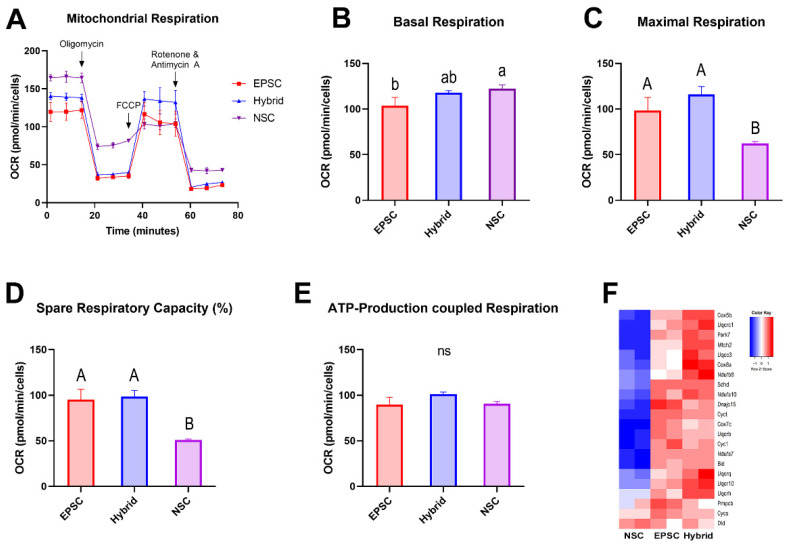
Metabolic remodeling in EPSC-NSC hybrid cells. (**A**) Measurement of oxygen consumption rate (OCR) in EPSCs, NSCs, and EPSC-NSC hybrid cells using Seahorse XFp analyzer. (**B**–**E**) Measurement of (**B**) basal respiration, (**C**) maximal respiration, (**D**) spare respiratory capacity (%), and (**E**) ATP-production-coupled respiration. Data are presented as the mean ± SD for *n* = 3 wells/group. ns, non-significant. ^A,B^ Uppercase and ^a,b^ lowercase indicate significant differences among different groups at *p* < 0.01 and *p* < 0.05. Data were analyzed using one-way ANOVA and Tukey’s post hoc with SAS^®^ software, version 9.4, (Institute of INC, Cary, NC, USA). (**F**) Heatmap of expression of genes categorized in ATP-synthesis-coupled electron transfer chain.

Metabolic remodeling in mitochondria represents an altered cellular state and plays a crucial role in the reprogramming process [38,41]. For example, in partially reprogrammed cells, the metabolic phenotype was distinct from that of pluripotent iPSCs, indicating a failure to acquire a bioenergetic system for maintaining pluripotency [42]. Therefore, we investigated the energy metabolism of EPSCs, hybrid cells, and NSCs using the Seahorse XFp analyzer to determine whether extended potential reprogramming by cell fusion entailed metabolic remodeling, as shown by changes in the molecular signature. To determine metabolic state, the oxygen consumption rate (OCR), which represents oxidative phosphorylation (OXPHOS) activity, was analyzed in EPSCs, hybrid cells, and NSCs (Figure 6A). The three cell types showed similar levels of basal respiration (Figure 6A,B). However, when cells were treated with FCCP, which maximizes mitochondrial respiration by collapsing the mitochondrial membrane potential, EPSCs showed nearly twice the maximal respiration compared to NSCs, indicating that the OXPHOS respiration capacity was more active in EPSCs than in NSCs (Figure 6A,C). As expected, the hybrid cells showed a level of maximal respiration almost equal to that of the EPSCs. In addition, EPSCs and hybrid cells exhibited a similar spare respiratory capacity (SRC) level, which was much higher than that of NSCs (Figure 6A–D). The ATP-coupled respiration rate was similar in all samples (Figure 6E). To confirm the correlation between energy metabolism and molecular signatures, we investigated the transcriptomes related to OXPHOS and the ATP-production-associated electron transfer chain (ETC). Consistent with the OCR results, EPSCs and hybrid cells showed higher levels of genes related to OXPHOS and ETC than NSCs (Figure 6F and Figure S7).

Next, the glycolytic activity of these cells was compared using the extracellular acidification rate (ECAR). In contrast to OCR, EPSCs exhibited higher levels of basal glycolysis than NSCs did (Figure S8A,B). Compensatory glycolysis after treatment with rotenone/antimycin A (Rot/AA), an inhibitor of electron transfer chain complexes I and II, was also higher in EPSCs than that in NSCs (Figure S8A,C). Additionally, EPSCs exhibited an upregulated glycolytic proton efflux rate (glycoPER) ratio compared with NSCs. Interestingly, the hybrid cells showed almost identical levels of EPSCs in basal glycolysis, compensatory glycolysis, and glycoPER. As glycoPER represents the glycolytic contribution to total extracellular acidification, these results indicate that EPSCs and hybrid cells actively use glycolysis for energy production (Figure S8A,D). Along with the results of the OCR analysis, these results indicate that EPSCs and hybrid cells actively use both OXPHOS and glycolysis for energy production and that this metabolic state can be faithfully remodeled toward EPSCs by fusion-induced reprogramming (Figure 6A–E and Figure S8A–D).

## 4. Discussion

In this study, we suggested that somatic cells, NSCs, could be directly reprogrammed to the extended pluripotent state by fusion with EPSCs. Reprogrammed hybrid cells exhibited extended pluripotential properties, including the expression of extended pluripotency markers, trophoblast lineage contribution in chimeric embryos, conversion into extraembryonic lineage stem cells (TSCs and XEN cells), and mitochondrial morphological remodeling and metabolic remodeling to the EPSC state. Furthermore, global gene expression patterns analyzed by RNA sequencing confirmed that the overall transcriptomic profile of hybrid cells was similar to that of EPSCs in terms of hierarchical clustering and GO terms, and they were distinguishable from traditional pluripotent stem cells (ESCs). Taken together, the somatic memory of NSCs was erased and extended pluripotency was established after fusion with EPSCs.

Fusion-induced reprogramming has previously been conducted using pluripotent stem cells such as ESCs, EGCs, ECCs, and induced PSCs (iPSCs), which can successfully reprogram somatic cells to a pluripotent state after cell fusion [19,20]. In the present study, we report for the first time that EPSCs can transfer extended pluripotency to somatic cells via cell fusion, although it remains as an open question if extended pluripotency can be transferred to terminal differentiated somatic cells such as fibroblasts. Furthermore, the reprogramming pathway toward extended pluripotency is yet to be elucidated. EPSCs, which were originally generated from eight-cell-stage embryos, could also be derived from ESCs or iPSCs by culturing in the LCDM condition for several passages, indicating reversibility between pluripotency and extended pluripotency [11]. Additionally, Liu et al. confirmed that human EPSCs could be directly established from fibroblasts by transduction of reprogramming factors (*Oct4*, *Sox2*, *Klf4*, and *c-Myc*) under the LCDM culture condition [43]. Therefore, reprogramming into extended pluripotency may be a stepwise process by which reprogrammed cells first acquire a pluripotent state and further acquire extended pluripotency during culture. However, it is not known whether the same stepwise process occurs in cell fusion reprogramming because it is a very fast and efficient reprogramming process; pluripotency marker activation and epigenetic reprogramming occur only 2 days after fusion [33]. Therefore, further research on the extended pluripotent reprogramming pathway is required.

In reprogramming toward pluripotency, chromosome-wide remodeling occurs 24–48 h after fusion, although epigenetic reprogramming takes more time [44]. In this study, we detected Oct4-GFP-positive hybrid cells 48 h after fusion with EPSCs. However, epigenetic reprogramming of extended pluripotency cannot be guaranteed with Oct4-GFP reactivation. Since DNA demethylation and reactivation of the X chromosome are the slowest reprogramming events [33,45], it is expected that these events may also occur relatively slowly during extended pluripotential reprogramming. Given that EPSCs have distinct epigenomic networks compared to naïve ESCs [11,46], observing time-course changes during cell-fusion-induced reprogramming may provide novel epigenetic and mechanistic insights into a totipotent state. For example, Mai et al. identified a novel transient reprogramming regulator, NKX3-1, using a cell-fusion-mediated heterokaryon model [47], suggesting that fusion-induced reprogramming could be a promising model for identifying early reprogramming factors [48].

By determining the developmental potency of hybrid cells, we showed that hybrid cells could contribute to the placenta and embryonic tissues. Placental contribution was observed in all 13.5 chimeric embryos (3/3), demonstrating a greater ability to contribute to placental tissues (Figure 3D and Figure S3A). In our previous studies, we showed that pluripotent hybrid cells formed by the fusion of somatic cells with ESCs could also develop into the placenta in chimeric embryos [17,49]. Therefore, not only the extraembryonic developmental potential, but also the tetraploidy of hybrid cells may contribute to the higher contribution ratio to the placenta.

However, differentiation efficiency toward trophoblast lineages in vitro was significantly less efficient. We showed that, although homogeneous XEN cell lines were easily established from hybrid cells in vitro, the conversion of hybrid cells into TSCs was relatively inefficient because homogenous TSCs were not induced, even after serial selection of TSC colonies. These results are consistent with previous reports that the formation of blastoids, or synthetic preimplantation embryos, using EPSCs resulted in a rare trophectoderm-like population compared to hypoblast- and epiblast-like contents [12,13]. In fact, the differentiation potential of EPSCs toward extraembryonic ectodermal lineages is highly diverse depending on the culture conditions [46,50], suggesting the importance of setting optimal differentiation conditions toward extraembryonic ectoderm.

We also demonstrated that the mitochondrial morphology and energy metabolism phenotype were remodeled to the state of EPSCs after cell-fusion-induced reprogramming. Mitochondrial shape and energy metabolism are cell-type-specific and contribute to the developmental potential of cells [51]. Therefore, changes in mitochondrial morphology and the consequent changes in metabolic patterns are highly dynamic in reprogramming and differentiation processes [51]. In this study, we showed that EPSCs and EPSC-NSC hybrid cells had a similar mitochondrial morphology, as well as bivalent bioenergetic activity, with higher levels of both OXPHOS and glycolysis than NSCs. Notably, EPSC-NSC hybrid cells exhibited an almost equivalent energy metabolism phenotype to EPSCs, but different from their fusion partners, NSCs, indicating faithful bioenergetic reprogramming. We expected that the OCR level would be higher in NSCs and the glycolytic level would be higher in EPSCs because pluripotent stem cells are more dependent on glycolysis than OXPHOS for energy metabolism [51,52]. However, EPSCs and EPSC-NSC hybrid cells showed a bivalent energy metabolism phenotype involving both OXPHOS and glycolysis. As this bivalent metabolic state is also observed in naïve pluripotent stem cells [41,53], EPSCs and naïve PSCs may share similar metabolic states. One possible reason for the bivalent energy phenotype of EPSCs and EPSC-NSC hybrid cells could be partially due to the inhibition of poly (ADP-ribose) polymerase-1 (Parp1) by minocycline hydrochloride (MiH) supplemented in the LCDM culture condition [11]. Parp1 is not only associated with various pathways underlying pluripotency and reprogramming, but is also considered a crucial regulator for the maintenance of EPSC potency [11,54]. Given that Parp1-knockout mouse ESCs showed altered expression of metabolism-related genes and preferential contribution to extraembryonic lineages [55], it is possible that Parp1 affects the bioenergetic metabolism, as well as the potency of EPSCs.

## 5. Conclusions

In the present study, somatic cells could be rapidly reprogrammed into an extended pluripotential state through cell-fusion-induced reprogramming with EPSCs. Fusion hybrid cells reconstructed EPSC-like characteristics, which entail transcriptional profile, acquisition of extraembryonic developmental potential, remodeling of mitochondrial morphology, and bivalent bioenergetic metabolism, with the erasure of somatic memory. In conclusion, we demonstrated that the extended pluripotential properties of EPSCs, including dual developmental potential and metabolic traits, could be faithfully transferred to somatic cells by cell-fusion-induced reprogramming.

## Data Availability

The datasets used and analyzed during the current study are available from the corresponding author upon reasonable request.

## Supplementary Materials

The following Supporting Information can be downloaded at: https://www.mdpi.com/article/10.3390/cells11203266/s1, Figure S1: In vitro random differentiation of embryonic germ layers from established EPSCs; Figure S2: The phase images of neural stem cells cultured under the N2B27-LCDM condition; Figure S3: Differentiational potency of EPSC-NSC hybrid cells in vivo and in vitro; Figure S4: GO:BP terms of differentially expressed genes among ESC, NSC, EPSC, and EPSC-NSC hybrid cells; Figure S5: Heatmap of expression of ectoderm and NSC marker genes; Figure S6: Heatmap of expression of genes related to placenta development; Figure S7: Heatmap of expression of genes categorized in ATP-synthesis-coupled electron transfer chain; Figure S8: Glycolytic function analysis of EPSCs, NSCs, and EPSC-NSC hybrid cells; Table S1: Primer sets used for real-time RT-PCR.
