# Supplementary material for: Developmental Potency and Metabolic Traits of Extended Pluripotency Are Faithfully Transferred to Somatic Cells via Cell Fusion-Induced Reprogramming
Source: Cells. 2022 Oct 17;11(20):3266. doi: 10.3390/cells11203266 (PMC9600027; doi:10.3390/cells11203266)
Supplement: Supplementary file 1 [file cells-11-03266-s001.zip › cells-1940433-supplementary.pdf]

Supplementary Materials

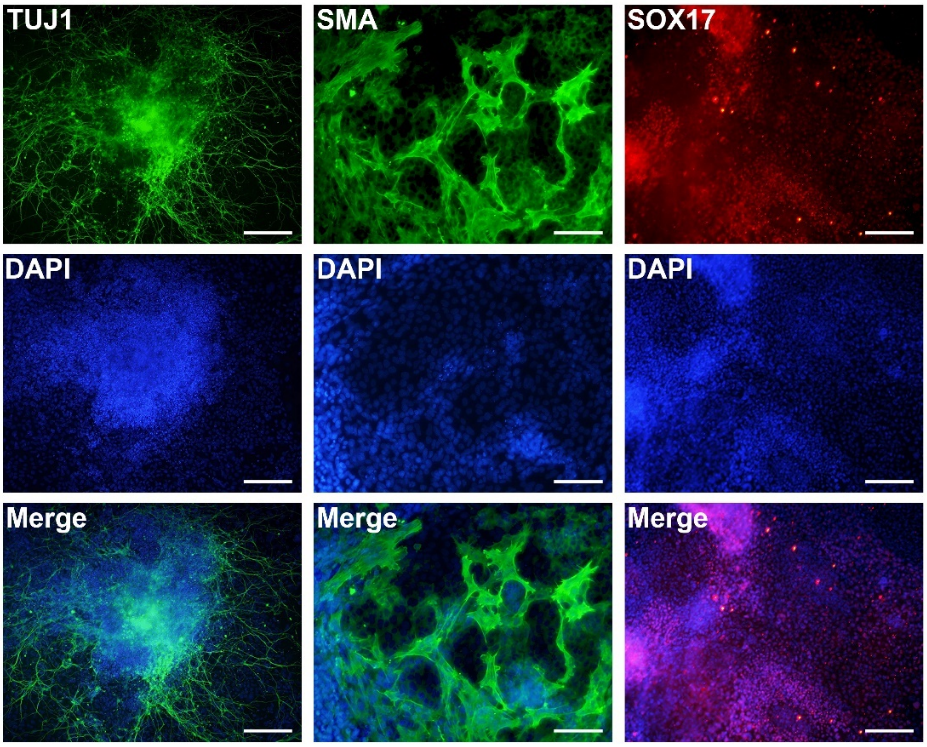

Figure S1. ***In vitro*** **random differentiation of embryonic germ layer from established EPSCs.** Immunofluorescence images of TUJ1 (ectodermal), SMA (mesodermal), and SOX17 (endodermal) markers in randomly differentiated EPSCs. Nuclei were stained by DAPI. Scale bars: 200 µm (TUJ1, SOX17), 100 µm (SMA)

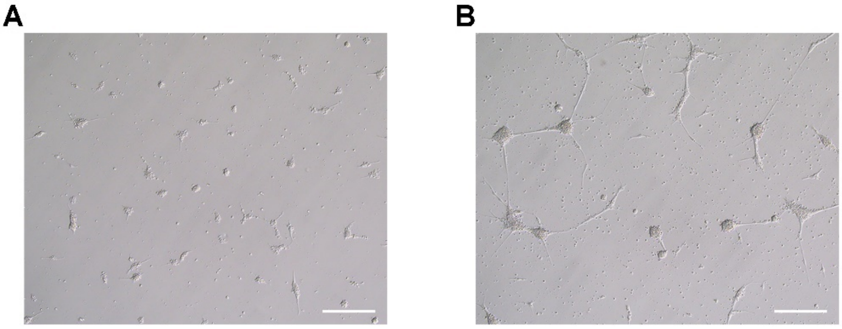

Figure S2. The phase images of neural stem cells cultured under the N2B27-LCDM condition.
(A) Bright-field images of NSCs on day 2 after being seeded in N2B27-LCDM medium. Scale bars: 200 μm (B) Bright-field images of NSCs on day 2 after changing culture condition to N2B27-LCDM at confluency > 70%. Scale bars: 200 μm

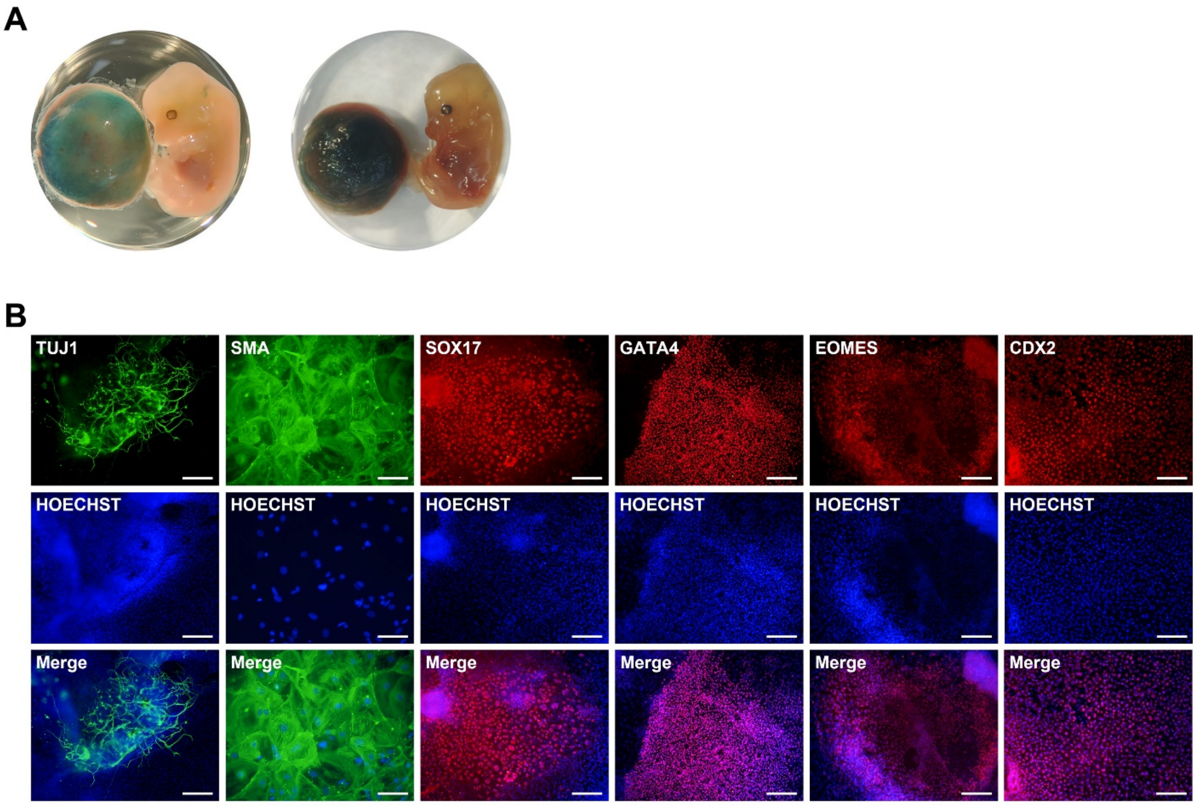

Figure S3. Differentiational potency of EPSC-NSC hybrid cells in vivo and in vitro
(A) X-gal staining results of chimeric E13.5 mouse embryo after transfer to the uterus of pseudo-pregnant mouse (B) Immunofluorescence images of TUJ1 (ectodermal), SMA (mesodermal), SOX17 (endodermal), GATA4 (extraembryonic endodermal), EOMES, and CDX2 (trophectodermal) markers in randomly differentiated hybrid cells. Nuclei were stained by Hoechst. Scale bars: 200 µm (TUJ1, SOX17, GATA4, EOMES, CDX2), 100 µm (SMA)

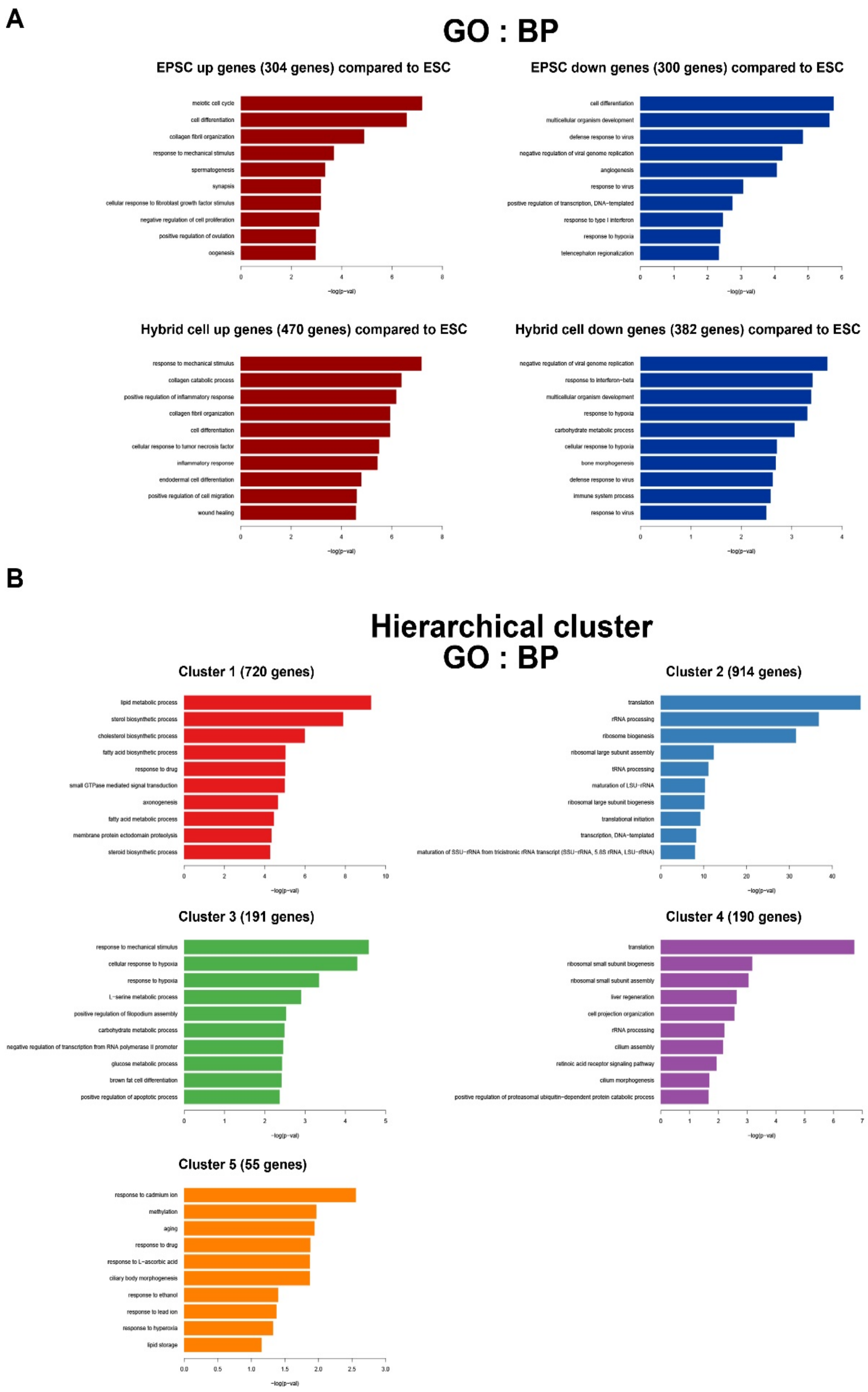
A
GO : BP
EPSC up genes (304 genes) compared to ESC
meiotic cell cycle
cell differentiation
collagen fibril organization
response to mechanical stimulus
spermatogenesis
synapsis
cellular response to fibroblast growth factor stimulus
negative regulation of cell proliferation
positive regulation of ovulation
oogenesis
−log(p−val)
EPSC down genes (300 genes) compared to ESC
cell differentiation
multicellular organism development
defense response to virus
negative regulation of viral genome replication
angiogenesis
response to virus
positive regulation of transcription, DNA−templated
response to type I interferon
response to hypoxia
telencephalon regionalization
−log(p−val)
Hybrid cell up genes (470 genes) compared to ESC
response to mechanical stimulus
collagen catabolic process
positive regulation of inflammatory response
collagen fibril organization
cell differentiation
cellular response to tumor necrosis factor
inflammatory response
endodermal cell differentiation
positive regulation of cell migration
wound healing
−log(p−val)
Hybrid cell down genes (382 genes) compared to ESC
negative regulation of viral genome replication
response to interferon−beta
multicellular organism development
response to hypoxia
carbohydrate metabolic process
cellular response to hypoxia
bone morphogenesis
defense response to virus
immune system process
response to virus
−log(p−val)
B
Hierarchical cluster
GO : BP
Cluster 1 (720 genes)
lipid metabolic process
sterol biosynthetic process
cholesterol biosynthetic process
fatty acid biosynthetic process
response to drug
small GTPase mediated signal transduction
axonogenesis
fatty acid metabolic process
membrane protein ectodomain proteolysis
steroid biosynthetic process
−log(p−val)
Cluster 2 (914 genes)
translation
rRNA processing
ribosome biogenesis
ribosomal large subunit assembly
tRNA processing
maturation of LSU−rRNA
ribosomal large subunit biogenesis
translational initiation
transcription, DNA−templated
maturation of SSU−rRNA from tricistronic rRNA transcript (SSU−rRNA, 5.8S rRNA, LSU−rRNA)
−log(p−val)
Cluster 3 (191 genes)
response to mechanical stimulus
cellular response to hypoxia
response to hypoxia
L−serine metabolic process
positive regulation of filopodium assembly
carbohydrate metabolic process
negative regulation of transcription from RNA polymerase II promoter
glucose metabolic process
brown fat cell differentiation
positive regulation of apoptotic process
−log(p−val)
Cluster 4 (190 genes)
translation
ribosomal small subunit biogenesis
ribosomal small subunit assembly
liver regeneration
cell projection organization
rRNA processing
cilium assembly
retinoic acid receptor signaling pathway
cilium morphogenesis
positive regulation of proteasomal ubiquitin−dependent protein catabolic process
−log(p−val)
Cluster 5 (55 genes)
response to cadmium ion
methylation
aging
response to drug
response to L−ascorbic acid
ciliary body morphogenesis
response to ethanol
response to lead ion
response to hyperoxia
lipid storage
−log(p−val)

Figure S4. GO:BP terms of differentially expressed genes between ESC, NSC, EPSC, and EPSC-NSC hybrid cell.

(A) Gene Ontology: Biological Process (GO:BP) analysis of Principle Component Analysis (PCA) between EPSCs and ESCs (top), hybrid cells and ESCs (bottom) (B) Gene Ontology: Biological Process (GO:BP) analysis of 1–6 enriched clusters of differentially expressed genes (DEGs) of ESCs, EPSCs, NSCs, and hybrid cells. Cluster 1 (upregulated in NSCs), cluster 2 (upregulated in EPSCs and hybrid cells), cluster 3 (downregulated in EPSCs and hybrid cells), cluster 4 (upregulated in ESCs), and cluster 5 (downregulated in ESCs) were analyzed.

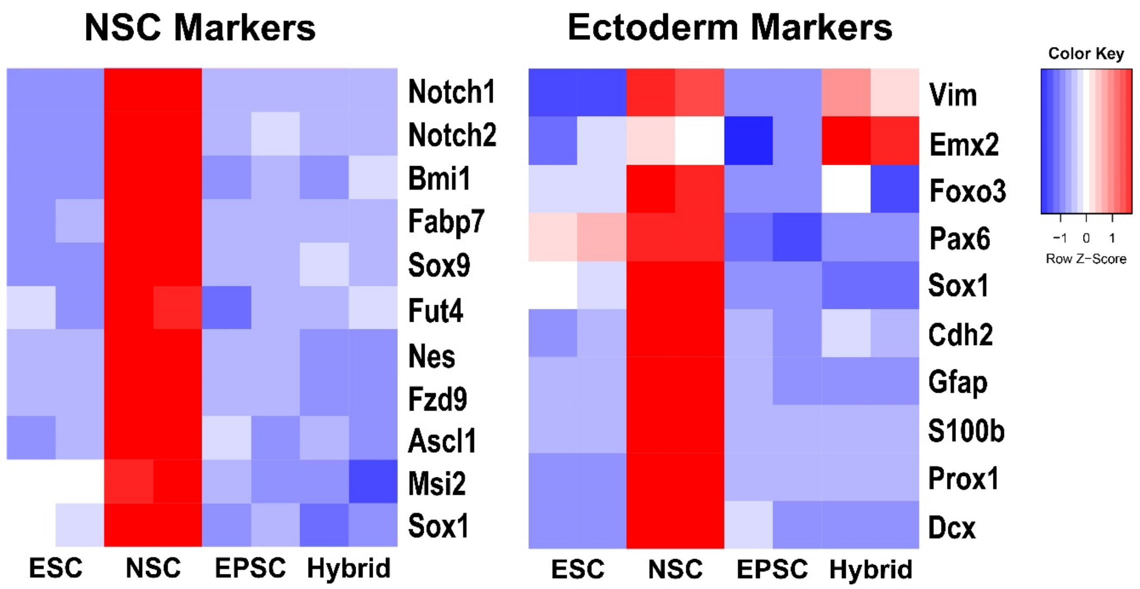

Figure S5. Heatmap of expression of ectoderm and NSC marker genes in ESCs, NSCs, EPSCs, and EPSC-NSC Hybrid cells

Heatmap of expression of ectoderm (left) and NSC (right) marker genes in ESCs, NSCs, EPSCs, and EPSC-NSC hybrid cells

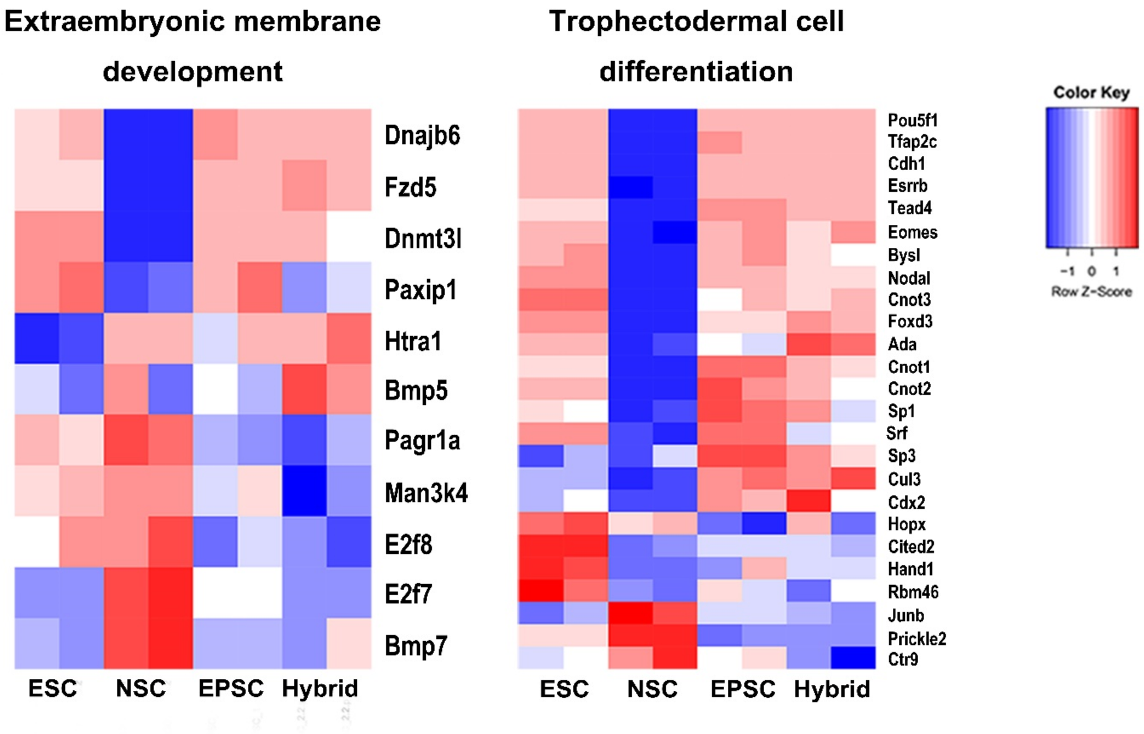

Figure S6. Heatmap of expression of genes related to placenta development in ESCs, NSCs, EPSCs, and EPSC-NSC Hybrid cells

Heatmap of expression of genes categorized in extraembryonic membrane development (left) and trophectodermal cell differentiation (right) in ESCs, NSCs, EPSCs, and EPSC-NSC hybrid cells

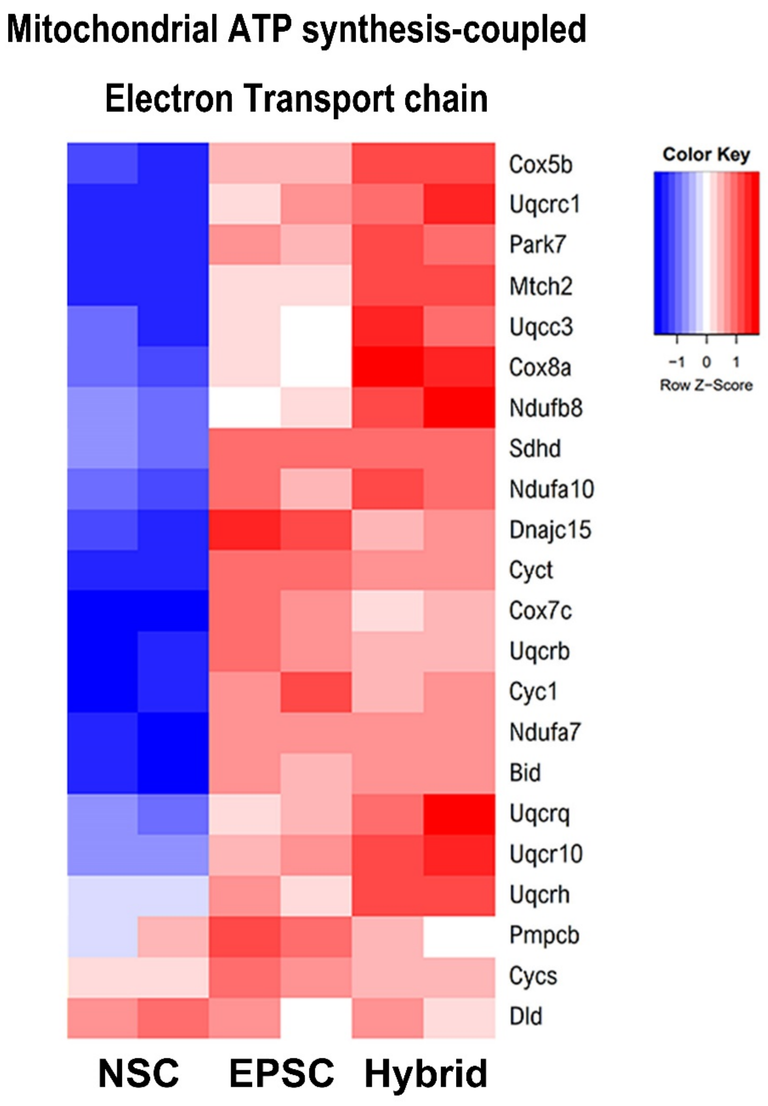

Figure S7. Heatmap of expression of genes categorized in mitochondrial ATP synthesis-coupled electron transport chain in NSCs, EPSCs, and EPSC-NSC Hybrid cells

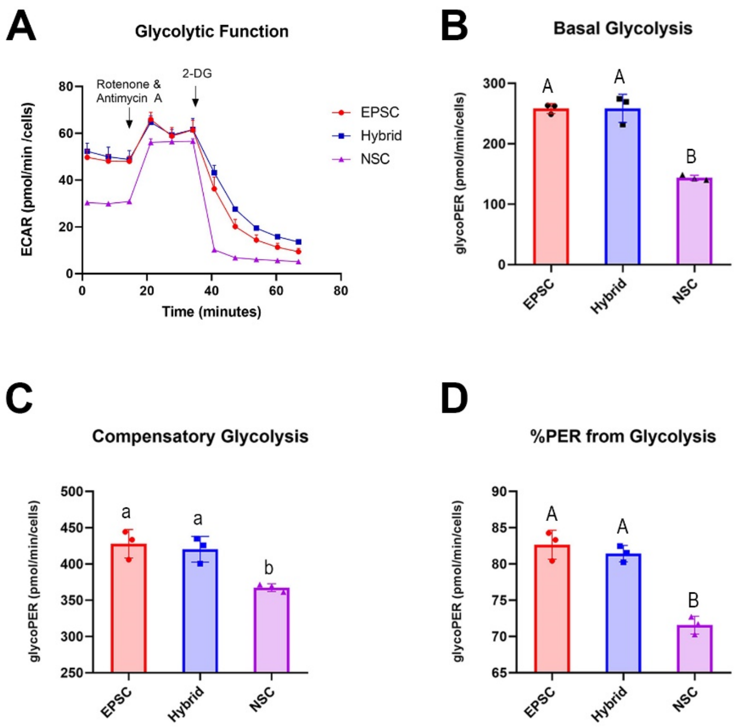

Figure S8. Glycolytic function of EPSC, NSC, and EPSC-NSC hybrid cell

(A) Measurement of extracellular acidification rate (ECAR) in EPSC, NSC, and EPSC-NSC hybrid cells. Measurement of (B) basal glycolysis, (C) compensatory glycolysis, and (D) % of proton efflux rate (PER) from glycolysis. Data are presented as mean ± SD for n=3 wells/group. $^{A\text{-}B}$ Uppercase and $^{a\text{-}b}$ lowercase indicate significant differences among different groups at $p<0.001$ and $p<0.01$. Data were analyzed using One-way ANOVA and Tukey's post hoc with SAS® software, version 9.4, (Institute of INC, North Carolina, USA).

Table S1. Primer sets used for real-time RT-PCR

Gene names	Sequence
Pou5f1_F	5' GAT GCT GTG AGC CAA GGC AAG 3'
Pou5f1_R	5' GGC TCC TGA TCA ACA GCA TCA C 3'
Nanog_F	5' CTT TCA CCT ATT AAG GTG CTT GC 3'
Nanog_R	5' TGG CAT CGG TTC ATC ATG GTA C 3'
Steap4_F	5' GGG AAG TCA CTG GGA TTG AAA A 3'
Steap4_R	5' CCG AAT AGC TCA GGA CCT CTG 3'
Tnc_F	5' ACG GCT ACC ACA GAA GCT G 3'
Tnc_R	5' ATG GCT GTT GTT GCT ATG GCA 3'
Csf1_F	5' ATG AGC AGG AGT ATT GCC AAG G 3'
Csf1_R	5' TCC ATT CCC AAT CAT GTG GCT A 3'
Bgn_F	5' TGC CAT GTG TCC TTT CGG TT 3'
Bgn_R	5' CAG GTC TAG CAG TGT GGT GTC 3'
Vcam1_F	5' AGT TGG GGA TTC GGT TGT TCT 3'
Vcam1_R	5' CCC CTC ATT CCT TAC CAC CC 3'
Postn_F	5' CGA TGT CTC GAA GCT GAG AG 3'
Postn_R	5' ACC ATG TGG CTG TGT AAG G 3'
Esrp1_F	5' CAA GCT GGG TTC GGA TGA GAA 3'
Esrp1_R	5' AGG TTT TCG GCG TCT ATT TTA GT 3'
Gapdh_F	5' GAT GCT GTG AGC CAA GGC AAG 3'
Gapdh_R	5' GGC TCC TGA TCA ACA GCA TCA C 3'
